# Chiral Chromatographic Analysis of Amino Acids with Pre-column Derivatization by o-Phthalaldehyde: Improving the Determination of Enantiomers Using Ion-Pair Reagents

**DOI:** 10.32607/actanaturae.27703

**Published:** 2025

**Authors:** N. V. Panin, I. V. Pirogov, D. T. Guranda, V. K. Švedas

**Affiliations:** Lomonosov Moscow State University, Belozersky Institute of Physicochemical Biology, Moscow, 119234 Russia; Lomonosov Moscow State University, Faculty of Chemistry, Moscow, 119234 Russia; Lomonosov Moscow State University, Faculty of Bioengineering and Bioinformatics, Moscow, 119234 Russia

**Keywords:** Chiral analysis of amino acids, o-phthalaldehyde, N-acetyl-L-cysteine, pre-column modification, HPLC conditions, ion-pair reagents, separation of diastereomeric isoindoles

## Abstract

The development of effective and accessible methods for the chiral analysis of amino acids is an important scientific and practical necessity. One of the most common and convenient techniques is the chromatographic determination of individual enantiomers of amino acids with preliminary conversion of enantiomers into diastereomers, which can then be separated on conventional achiral columns. We have shown that by adding ion-pair reagents to the eluent and varying their structure, one can regulate the efficiency of a chiral amino acid analysis based on the chromatographic determination and resolution of the diastereomeric isoindoles obtained upon pre-column derivatization of amino acids by o-phthalaldehyde in the presence of N-acetyl-L-cysteine. The use of ion-pair reagents allows one to achieve a better resolution of diastereomeric isoindole peaks, while the analysis time can be reduced by increasing the ionic strength. Hence, adding ionpair reagents and optimizing the mobile phase composition are useful approaches in the engineering of an amino acid chiral analysis, along with the synthesis of new chiral SH compounds and the choice of stationary phases.

## INTRODUCTION

The need to determine individual enantiomers in the total content of amino acids and other amino compounds is an important undertaking both in fundamental research and medical diagnostics, as well as in the characterization of raw materials and manufactured products in the pharmaceutical and food industries [1, 2, 3, 4, 5, 6, 7, 8, 9, 10, 11, 12]. Significant attention is also paid to the stereoisomerism of amino acids in the environment under prebiotic conditions when studying the origin of life [13, 14]. The scale and complexity of the problems related to determining the individual enantiomers of amino acids in complex mixtures has increased significantly in recent years, as highly effective methods of chiral metabolomics are pursued [15, 16, 17]. Methods for an efficient, rapid, and widely accessible chiral analysis of amino acids as building blocks of physiologically active compounds and markers of various pathological processes are sorely needed in systematic research into living systems in the postgenomic era. Chromatographic methods – primarily high-performance liquid chromatography (HPLC) with pre-column derivatization of enantiomers into diastereomers which can then be separated on conventional achiral columns – are the most widely used means for the chiral analysis of amino compounds. One of the most accessible, convenient, and effective methods is pre-column modification of amino groups by o-phthalaldehyde (OPA) and a chiral derivatizing SH reagent (CDR) [18, 19]. This modification of amino compounds occurs quite quickly: unlike the method utilizing ninhydrin, there is no need to increase the temperature; the resulting diastereomeric isoindoles are usually stable under the conditions of the analysis and are characterized by different retention times on standard HPLC columns. Isoindoles have a characteristic absorption maximum at 340 nm and a molar absorption coefficient of 6,000 M-1cm-1 and are good fluorophores, which allows one to determine the femtomoles of the amino compounds using fluorescent detectors if the sensitivity of spectrophotometric measurements is insufficient [20, 21].

**Fig. 1:**
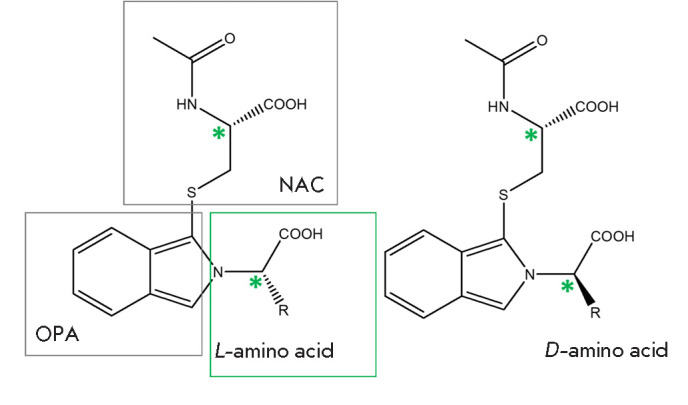
Diastereomeric isoindole adducts formed upon derivatization of amino acids by OPA and a chiral thiol (N-acetyl-L-cysteine). R is the side chain of the amino acid

**Fig. 2:**
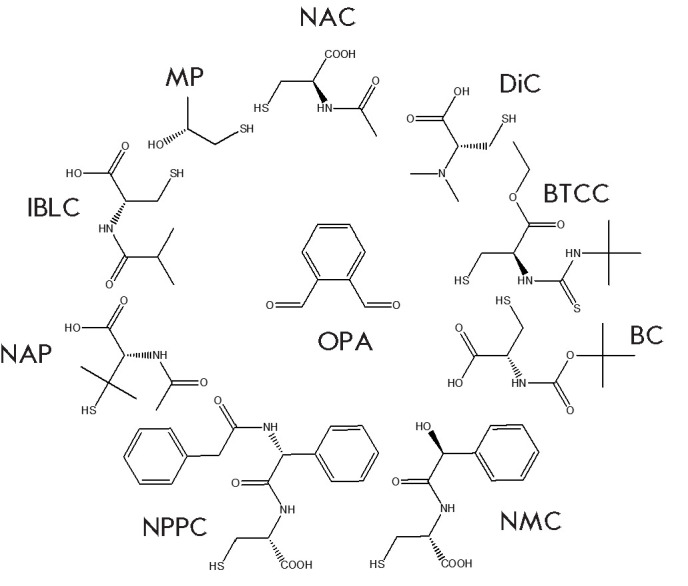
Chiral thiols used for pre-column derivatization by OPA

In the best-known version of this method, a very cheap and accessible CDR, N-acetyl-L-cysteine (NAC) (Fig. 1) [18], is used to determine the enantiomers of α-amino acids. However, it remains impossible to achieve the required resolution for all the compounds belonging to this class, as well as for other amino compounds. NAC analogs have been proposed to improve the efficiency of this procedure (Fig. 2): N-isobutyryl-L-cysteine [22], ethyl ester of N-tertbutylthiocarbamoyl-L-cysteine (BTCC) [23], BocL-cysteine, N-acetyl-(R)-penicillamine (NAP) [24], N-phenylacetyl-(R)-phenylglycyl-L-cysteine (NPPC) [25], N-(R)-mandelyl-L-cysteine (NMC) [26, 27], N,Ndimethyl-L-cysteine (DiC) [28], 1-mercapto-2-propanol (MP) [29] and other SH reagents.

The use of SH reagents that have various structures allows one to significantly broaden the application range of the method used to determine the enantiomers of a wide range of amino compounds: amino acids, primary amines, and amino alcohols. Thus, whereas it is possible to achieve an acceptable resolution only for a small number of aliphatic amines for the conventional NAC, when using R-NMC, which contains two chiral centers and a large number of intramolecular contacts, it is possible to pinpoint a number of amine and amino alcohol enantiomers, including those that are not resolved even on chiral columns [26]. However, most of the proposed SH compounds are not readily available, since they are not commercial reagents and are mainly used by the research groups that have proposed them to solve a limited range of analytical problems. This fact makes any assessment of the prospects for a wider application of novel SH reagents rather challenging. Along with creating new CDRs, engineering the analytical process per se (the stationary and mobile phases, as well as the analytical conditions) can be an alternative approach to improving the efficiency of chromatographic determination of the diastereomers of amino compounds. Adding ion-pair reagents (IPRs) is one of the unexplored possibilities of mobile phase engineering in reversed-phase HPLC for a more efficient resolution of diastereomers. The experience of using ionpair reagents to improve the chromatographic resolution of structurally similar compounds demonstrates that this approach is rather promising [30, 31, 32]. The introduction of an IPR carrying a charged functional group and nonpolar fragments (alkyl radicals) can increase analyte retention on a chromatographic column through reagent sorption on the surface of the reversed-phase adsorbent and changes in the interaction with the analyte. The resolution of organic acids was thus improved by adding quaternary ammonium salts in the mobile phase (tetrabutylammonium bromide (TBA) being one used most commonly) [30].

The feasibility of using ion-pair reagents for a more efficient chromatographic resolution of diastereomers obtained upon pre-column derivatization of α-amino acids by OPA and the chiral thiol NAC was investigated for the first time in this study.

## EXPERIMENTAL

**Reagents**

o-Phthalaldehyde (OPA; 99%, Koch Light, England), N-acetyl-L-cysteine (NAC; 99%, AppliChem, Germany), phenylalanine (Reakhim, Russia), glutamate (Aurat, Russia), leucine, asparagine and arginine (Reanal, Hungary), tetrabutylammonium bromide (TBA), N,N,N-trimethyloctylammonium bromide (OTMA) (ABCR GmbH, Germany) were used in this study. Buffer components, acids, and alkalis were the domestic products of highest purity available; methanol (PanReac, Spain) was of pure, for the analysis, grade; acetonitrile (Kriokhrom, Russia) was of HPLC grade.

**HPLC analysis**

The HPLC analysis was performed in a Perkin Elmer 200 Series chromatographic system: Kromasil Eternity 5-C18 4.6 × 250 mm reversed-phase chromatography column; injection volume, 10 μL; flow rate, 1 mL/min. The two-channel system operation mode was used to prepare the mobile phase with a given acetonitrile concentration: channel A – 5 mM phosphate buffer pH 6.8, 10% acetonitrile; channel B – 5 mM phosphate buffer pH 6.8, 80% acetonitrile.

When studying the effect of IPR, elution was performed in the isocratic mode using only channel A. TBA or OTMA at a final concentration of 5 mM and, in different experiments 15%, 20%, or 30% acetonitrile were added to the mobile phase based on 5 mM phosphate-buffered saline pH 6.8. HPLC analysis with the addition of an IPR was performed after preliminary column equilibration for 1 h to ensure maximal reproducibility of the results. When studying the effect of the ionic strength, NaCl was additionally added in channel A at a final concentration of 50 mM. Isoindole diastereomers were detected spectrophotometrically at 340 nm. The absorption intensity was measured in μV (arbitrary units, a.u.). TotalChrom Navigator 6.3.2 was used for HPLC system control and data processing.

**Pre-column derivatization**

Derivatization of primary amino groups was performed automatically using the Derivatization software function of the autosampler as follows: 20 μL of a 5 mM amino acid solution, 20 μL of a 10 mM methanolic OPA solution, and 20 μL of a 40 mM NAC solution were successively added to a cell containing 500 μL of 0.1 M borate buffer, pH 9.6, using an autosampler needle, followed by stirring of the reaction mixture with an autosampler needle in the automatic mode. The mixture prepared in this way was left to rest for 15 min; 50 μL of a 50 mM IPR solution was then added (if necessary) for preliminary equilibration of the system and analyzed by HPLC.

## RESULTS AND DISCUSSION

**Resolution of amino acid enantiomers after pre-column derivatization**

**Fig. 3:**
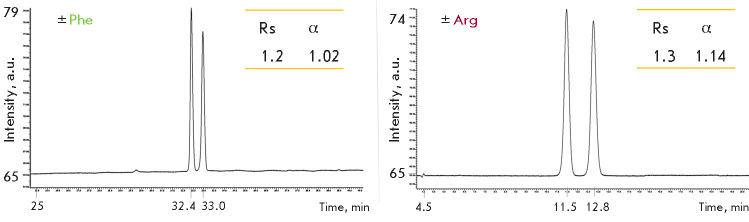
Chromatograms of isoindole derivatives of phenylalanine and arginine enantiomers obtained upon pre-column derivatization by OPA in the presence of NAC. Gradient elution mode: 5 mM phosphate-buffered saline, pH 6.8, 0–10 min 10% CH_3_ CN, 10–60 min 10–40% CH_3_ CN

The series of enantiomers to be resolved included α-amino acids with different physicochemical side chain characteristics: glutamic acid, arginine, phenylalanine, leucine, and asparagine. At the first step, chromatographic analysis after pre-column derivatization by OPA and NAC was carried out on a conventional achiral C18 column at a neutral pH 6.8 in the gradient elution mode (0–10 min: 10% CH_3_ CN, 10–60 min: 10–40% CH_3_ CN). Under these conditions, resolution of diastereomeric isoindole derivatives was observed only for arginine and phenylalanine (Fig. 3); therefore, ion-pair reagents were added to the mobile phase at the next step to improve the resolution of other analytes.

**The effect of adding ion-pair reagents**

**Fig. 4:**
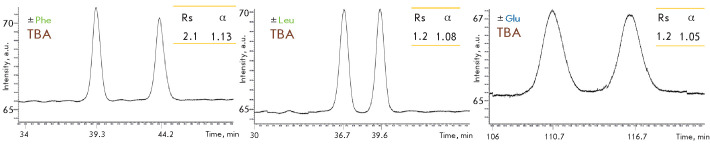
The effect of adding an IPR to the mobile phase during a chromatographic analysis of isoindole derivatives obtained upon precolumn derivatization of phenylalanine, leucine, and glutamate enantiomers by OPA in the presence of NAC. Isocratic mode: 5 mM phosphate-buffered saline, pH 6.8, 5 mM TBA, 20% CH_3_ CN (in the case of Glu), 30% CH_3_ CN (in the case of Leu and Phe)

Quaternary ammonium salts with different alkyl substituents were chosen as IPRs, since the isoindole adducts to be resolved upon chromatographic analysis at pH 6.8 carry two negatively charged carboxyl groups. Addition of tetrabutylammonium bromide (TBA) as an IPR to the eluent leads to chromatographic resolution of the isoindole derivatives of leucine and glutamic acid enantiomers, as well as to a significant improvement in the case of phenylalanine (Fig. 4). Efficient chiral analysis of glutamate enantiomers is achieved at a lower concentration of the organic solvent in the eluent.

Interestingly, addition of this IPR does not improve the chromatographic resolution of arginine, since the formation of an ion-pair associate with TBA is apparently hindered by the presence of a positively charged guanidine group in the amino acid side chain, and effective chiral analysis of arginine can be done in the “normal” mode (see Fig. 3).

**The effect of the structure of the ion-pair reagent**

**Fig. 5:**
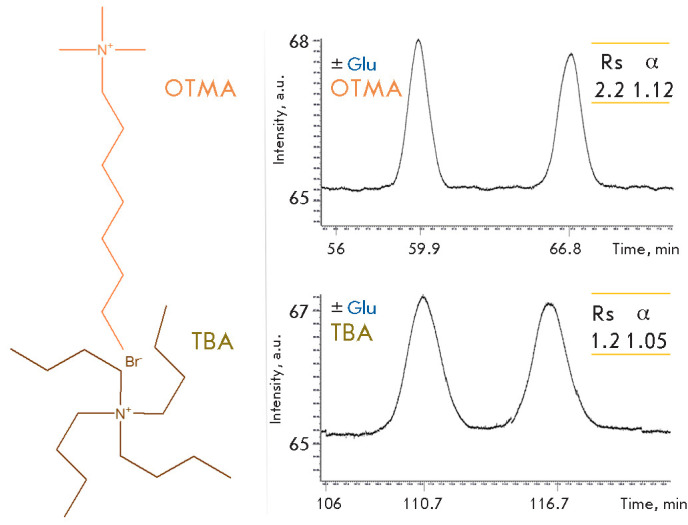
The effect of symmetric (TBA) and asymmetric (OTMA) IPR on the chromatographic resolution of isoindole derivatives obtained upon precolumn derivatization of glutamic acid enantiomers by OPA in the presence of NAC. Isocratic mode: 5 mM phosphate-buffered saline, pH 6.8, 20% CH_3_ CN, 5 mM IPR

An asymmetric IPR, N,N,N-trimethyloctylammonium bromide OTMA (Fig. 5), was used along with symmetric TBA when studying the effect of the IPR structure on the resolution of isoindole derivatives of amino acid enantiomers.

Compared to TBA, addition of OTMA reduces the elution time of isoindole derivatives of glutamic acid, phenylalanine, and leucine. For the negatively charged glutamic acid, in contrast to neutral phenylalanine and leucine, the analysis time is reduced and the resolution is improved (Fig. 5). This impact can be explained by the fact that when asymmetric OTMA is added to the mobile phase, the long aliphatic radical becomes deeply and firmly bound by the C18 stationary phase and a classical strong anion exchanger is formed [33]: the resolution of anions occurs on it via a competing mechanism. The ion-exchange mechanism of anion sorption is proven to be involved by the fact that retention strongly depends on the ionic strength of the eluent, which is the main mean of regulating the strength of anion retention.

**The effect of the ionic strength**

**Fig. 6:**
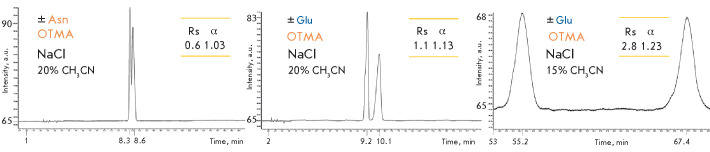
Chromatograms of isoindole derivatives of asparagine and glutamic acid enantiomers obtained upon pre-column derivatization by OPA in the presence of NAC. Isocratic mode: 5 mM phosphate-buffered saline, pH 6.8, 5 mM OTMA, 50 mM NaCl, 20% CH_3_ CN (left and center) and 15% CH_3_ CN (right)

In order to understand how the ionic strength influences the impacts of the addition of IPRs to the mobile phase, 50 mM of NaCl was added to the eluent containing OTMA. The study showed that an increase in ionic strength significantly reduces the retention time of diastereomeric isoindoles and shortens the analysis time (Fig. 6, left and center). Under these conditions, the isoindole derivatives of polar-uncharged asparagine also start to separate (Fig. 6, left). Improved resolution can be achieved at lower concentrations of the organic solvent in the eluent (compare Fig. 6, right and center).

**Fig. 7:**
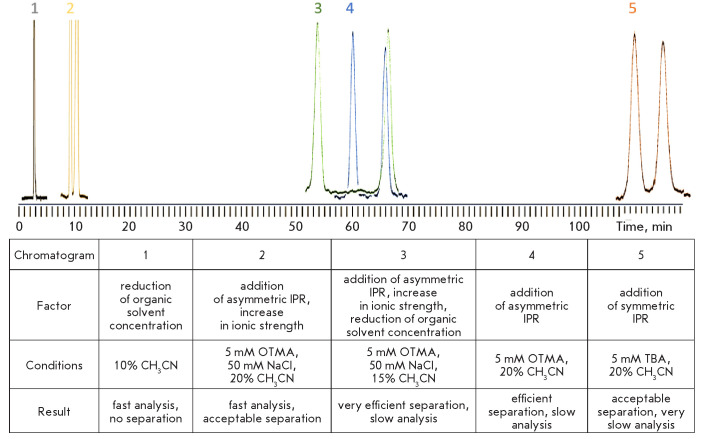
The influence of various factors on the efficiency of chromatographic resolution and the time of analysis of isoindole derivatives of glutamic acid enantiomers obtained upon pre-column derivatization by OPA in the presence of NAC. Isocratic mode: 5 mM phosphate-buffered saline, pH 6.8

Our experiments showed that by adding IPRs to the eluent and varying their structure, one can regulate the efficiency of a chiral amino acid analysis based on the chromatographic determination and resolution of diastereomeric isoindoles obtained upon pre-column derivatization of amino acids by OPA in the presence of NAC. Better resolution of the peaks of the identified isoindoles of phenylalanine, leucine, glutamic acid and asparagine can be achieved by using IPRs. Although it is accompanied by longer analyte retention on the column, the analysis time can be reduced by using an asymmetric IPR (OTMA) and increasing the ionic strength of the eluent. Figure 7 shows the characteristic effect of various factors on the efficiency of the chromatographic resolution of the isoindole derivatives of amino acid enantiomers using glutamic acid.

## CONCLUSIONS

The development of effective and accessible methods for the chiral analysis of amino acids is an important issue in scientific research, medical diagnostics, and the characterization of a wide range of products manufactured by the pharmaceutical and food industries. Chromatographic determination of the individual enantiomers of natural and synthetic amino acids using achiral columns after pre-column derivatization of samples by OPA in the presence of chiral SH compounds is one of the most common and convenient techniques used today. This study showed that we can achieve better resolution of the peaks of the determined diastereomeric isoindoles using ion-pair reagents. By varying the structure of the ion-pair reagent and increasing the ionic strength of the mobile phase, one can achieve a more efficient resolution of diastereomers and shorten the analysis time. Hence, addition of ion-pair reagents to the mobile phase is a useful approach in engineering a chiral amino acid analysis, along with the synthesis of chiral SH compounds and the choice of stationary phases.

## Abbreviations

OPA: o-phthalaldehyde
NAC: N-acetyl-L-cysteine
HPLC: high-performance liquid chromatography
IPR: ion-pair reagent
TBA: tetrabutylammonium bromide
OTMA: N,N,N-trimethyloctylammonium bromide
CDR: chiral derivatizing SH reagent
BTCC: N-tert-butylthiocarbamoyl-L-cysteine ethyl ester
NAP: N-acetyl-D-penicillamine
NMC: N-(R)-mandelylL-cysteine
DiC: N,N-dimethyl-L-cysteine
IBLC: N-isobutyryl-L-cysteine
NPPC: N-phenylacetyl-(R)- phenylglycyl-L-cysteine
MP: 1-mercapto-2-propanol
BC: Boc-L-cysteine
a.u.: arbitrary units
